# Patient‐reported outcome measures compared to professional dental assessments of monolithic ZrO_2_ implant fixed dental prostheses in complete digital workflows: A double‐blind crossover randomized controlled trial

**DOI:** 10.1111/jopr.13589

**Published:** 2022-08-23

**Authors:** Aiste Gintaute, Nicola U. Zitzmann, Urs Brägger, Karin Weber, Tim Joda

**Affiliations:** ^1^ Department of Reconstructive Dentistry University Center for Dental Medicine, University of Basel Basel Switzerland; ^2^ Department of Reconstructive Dentistry and Gerodontology, School of Dental Medicine University of Bern Bern Switzerland; ^3^ Clinic of Reconstructive Dentistry, Center of Dental Medicine University of Zurich Zurich Switzerland

**Keywords:** clinical research, dental implant, digital dentistry, fixed dental prosthesis (FDP), patient satisfaction

## Abstract

**Purpose:**

This double‐blind randomized controlled trial analyzed patient‐reported outcome measures in terms of subjective patient satisfaction compared to objective dental evaluation of prosthetic treatment with 3‐unit monolithic zirconium dioxide implant fixed dental prostheses (iFDPs) in 3 digital workflows.

**Material and methods:**

Twenty patients were restored with 3 iFDPs each on Straumann TL‐implants with 2 completely digital workflows using different intraoral optical scanning systems with model‐free fabrication of the restoration (Trios 3/3Shape [Test‐1]; Virtuo Vivo/Straumann [Test‐2]), and mixed analog‐digital workflow with conventional impressions and digitized gypsum casts (Impregum/3M Espe [Control]). The order of impression‐taking and the prosthetic try‐in were randomly allocated. Sixty iFDPs were compared for patient satisfaction and dental evaluation using ANOVA.

**Results:**

For iFDP evaluation, patients generally provided more favorable ratings than dental experts, regardless of the workflow. ANOVA revealed no significant difference for overall satisfaction when comparing Test‐1, Test‐2, or Control, either for patients (f‐ratio: 0.13; *p* = 0.876) or dentist (f‐ratio: 1.55: *p* = 0.221). Secondary, patients clearly favored the digital impression workflows over the conventional approach (f‐ratio: 14.57; *p* < 0.001). Overall, the 3Shape workflow (Test‐1) received the highest scores for all analyses.

**Conclusions:**

The different digital workflows demonstrated minor influence on the subjective and objective evaluation of the monolithic zirconium dioxide iFDPs in nonesthetic regions; however, the dentist may significantly increase patient satisfaction by choosing intraoral scanning instead of conventional impressions. The dentist has to consider individual patients’ needs to fulfill their expectations for a personalized solution.

In the past, the choice of patient therapy was determined by the dentist, often in consultation with the dental technician for choice of materials. Over time, this monodirectional decision‐making relationship between clinician and patient has changed.^1^ The advent of evidence‐based dentistry, coupled with widespread access to information on dental treatment options on the internet, has shifted the focus towards providing transparent patient information that explains the advantages and disadvantages of different treatment options, thereby directly involving the patient in the treatment planning process.^2^ Treatment can only be successful if both the objectifiable parameters on the part of the dentist and the subjective perception and satisfaction of the patient are in harmony.^3^, ^4^ In line with this change in clinical patient management, dental research has also evolved to become more patient‐centric. In addition to the classic clinical outcomes used in prosthodontic research, such as the precision of dental restorations, patient‐reported outcome measures (PROMs) have been integrated as primary and secondary parameters in many clinical studies.^5^, ^6^, ^7^, ^8^, ^9^, ^10^, ^11^ New insights will promote patient engagement in treatment decision‐making, and should therefore improve overall patient satisfaction by communicating mutual expectations from the outset.^12^, ^13^ Patients' needs can be better perceived, and future research can be directed toward what is in the best interest of patients—rather than research for its own sake.

The question of patient‐specific benefits is currently being raised in the context of the ubiquitous increase in digitization.^14^ Do dental workflows have to be digital because it is modern and trendy these days? Do patients expect this from the dental profession or is it triggered by the media and industry? The continuous development in dental processing ensures new opportunities in the field of fixed prosthodontics in a complete virtual environment without any physical model situations. Particularly, the challenge in the complete digital workflow is the virtual definition of a functionally correct occlusion and the further fabrication without physical models. For treatment with implant fixed dental prostheses (iFDPs), recent randomized controlled trials could demonstrate superiority of complete digital implant workflows over conventional analog workflows in terms of clinical^15^, ^16^, ^17^, ^18^ as well as economic outcomes.^19^, ^20^, ^21^, ^22^ However, there is limited clinical evidence for the impact of digital versus conventional workflows on PROMs in patients treated with multiunit monolithic zirconium dioxide (ZrO_2_) iFDPs.^23^

The aim of this double‐blind randomized controlled trial (RCT) with a crossover design was to analyze implant prosthetic treatment with monolithic ZrO_2_ iFDPs performed using 2 proprietary completely digital workflows (Test‐1 and Test‐2) and 1 mixed analog‐digital workflow (Control) in terms of PROMs compared with professional dental evaluation. The null hypotheses were that (1) subjective patient satisfaction would be similar when comparing iFDPs prepared using these 3 workflows; (2) patient satisfaction would not correlate with objective dental evaluation; and (3) patients would not have any preferences among the 2 IOS and the conventional implant impressions.

## MATERIAL AND METHODS

### Trial design

This investigation is part of a triple‐arm double‐blind RCT with a crossover design analyzing different outcomes on the same population.^24^, ^25^ The present manuscript reports on subjective patient satisfaction and professional dental assessments for prosthetic treatment with 3‐unit monolithic ZrO_2_ iFDPs in the posterior region. The Ethics Committee Basel, Switzerland (EKNZ‐ID 2019‐00706) approved the study protocol; and it was registered at ClinTrials.gov (NCT04029025). This RCT was conducted in compliance with the study protocol, the current version of the Declaration of Helsinki, ICH‐GCP, and all national legal and regulatory requirements. Included patients provided an informed consent. No changes were made to methods after trial commencement, and the RCT followed the CONSORT 2010 statements (http://www.consort‐statement.org/consort‐2010).

**FIGURE 1:**
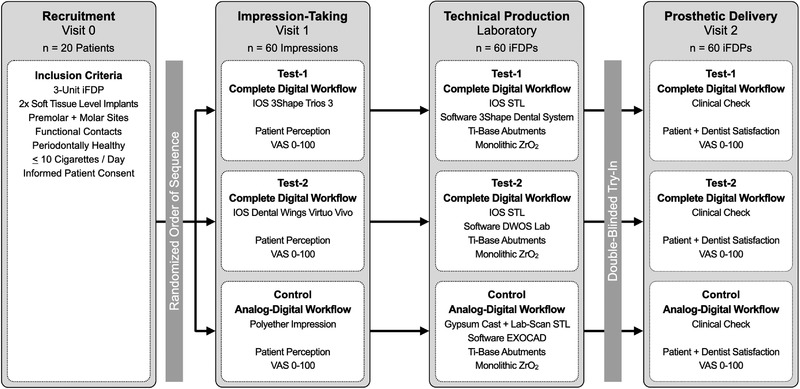
Study flowchart: triple‐armed, double‐blinded, double‐randomized controlled trial with crossover design. iFDPs = implant fixed dental prostheses, IOS = intraoral optical scanning, VAS = visual analogue scale, ZrO_2_ = zirconium dioxide.

The detailed trial setting has been described previously.^24^, ^25^ Inclusion criteria were 2 dental implants intended for a 3‐unit iFDP (Tissue Level Implant System RN/WN, Institut Straumann AG, Basel, Switzerland) with baseline at the start of the prosthetic therapy. Briefly, 20 study patients received 3 iFDPs each, resulting in a total of 60 restorations. All iFDPs were CAD‐CAM‐processed out of monolithic ZrO_2_ (VITA YZ ST Super Translucent Multicolor, Bad Säckingen, Germany) bonded to pre‐fabricated titanium abutments (Variobase, Institut Straumann AG, Basel, Switzerland) (Fig 1):

- Test‐1 Group “Complete Digital Workflow‐1” (3Shape, Copenhagen, Denmark):
  IOS Trios 3 + Dental System Lab‐Software;
- Test‐2 Group “Complete Digital Workflow‐2” (Dental Wings Inc., Montreal, Canada):
  IOS Virtuo Vivo + DWOS Lab‐Software;
- Control Group “Mixed Analog‐Digital Workflow”:
  Polyether Impression / Gypsum Cast / Lab‐Scan + Exocad Lab‐Software.

The sequence of the worksteps, whether beginning with Test‐1, Test‐2, or Control, as well as the order of sequence for the restorations’ try‐in, were randomly assigned per study patient using the envelope‐technique. The principal investigator (TJ) performed the random allocation sequence. Both the clinical operator and the study patient were unaware at the time of prosthetic try‐in, which of the iFDPs were produced by which of the 3 different groups. Clinical worksteps were performed by 1 experienced dentist (KW) and observed by 1 neutral spectator (AG).

### Outcomes

**FIGURE 2:**
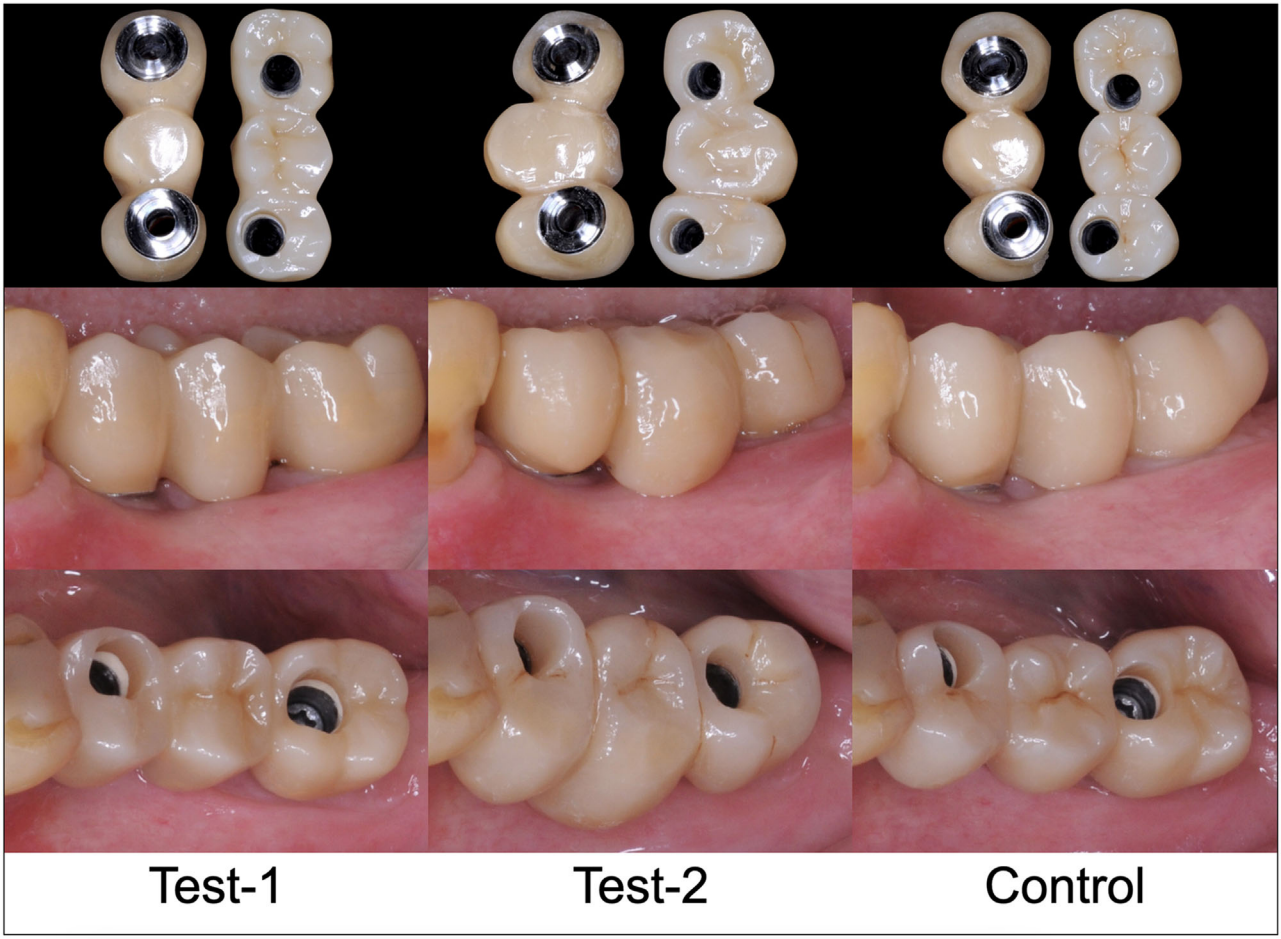
Study patient #06: randomized order of double‐blind clinical try‐in of monolithic ZrO_2_ iFDPs produced by Test‐1 (IOS TRIOS 3 + Dental System Lab‐Software [3Shape]), Test‐2 (IOS Virtuo Vivo + DWOS Lab‐Software [Dental Wings]), and Control (Polyether Impression, Gypsum Cast, Lab‐Scan + Exocad Lab‐Software [EXOCAD]). iFDPs = implant fixed dental prostheses, ZrO_2_ = zirconium dioxide.

The primary outcome was defined as the blinded evaluation of 3 monolithic ZrO_2_ iFDPs by the patient (subjective satisfaction) and the dentist (objective evaluation) at the time of prosthetic try‐in (Fig 2). Opinions on overall satisfaction with the iFDP were assessed with visual analog scale questionnaires (VAS; range 0‐100). In addition, a correlation analysis was performed comparing patient and dentist opinions.

As a secondary outcome, after the impression session, all patients were asked about their perceptions related to the clinical handling and convenience of the implant impression workflows of Test‐1, Test‐2, and Control. Six questions per workflow addressed treatment time, self‐perception of the applied impression procedures in terms of convenience, anxiety, taste, nausea sensation, and possible pain during or immediately after impression‐taking procedures. The questions on patient‐reported outcome measures were adapted from a previously published study that compared IOS with conventional impression taking for treatment with single‐unit implant restorations in posterior sites.^26^

### Statistics

At the time of study plan development, no trials were available analyzing PROMs for multiunit iFDPs. Therefore, a statistical power analysis was performed based on preliminary investigations for treatment with single‐unit implant crowns using VAS questionnaires ranging from 0 to 100.^26^ It was assumed that Test workflows, representing digital impressions, would have a satisfaction rate of VAS = 79 (SD ±15), whereas a satisfaction rate of VAS = 54 would be expected for the Control workflow with conventional implant impression and comparable range of standard deviations.^26^ A significant difference between the workflows could be determined with 12 individuals per group with a statistical power of 80% and a level of significance of 0.05. Normal distribution was assumed; therefore, the inclusion of 20 study patients were defined as a precaution in case of a higher SD level.

Statistical analysis allowed assessment of differences in patient satisfaction and dentist evaluation between the 3 workflows. Calculations were made with the computer program “Software R” (version 4.2.0). ANOVA tests were used for all comparisons. The level of significance was set at *α* = 0.05. The Tukey's HSD (honestly significant difference) procedure was used for pairwise comparisons within ANOVA data. The F‐statistic reported whether there is an overall difference between the sample means. Tukey's HSD test determined between which of the various pairs of means, if any, demonstrated a significant difference.

## RESULTS

A total of 20 study patients received the intended treatment and were double‐blinded analyzed for primary and secondary outcomes. After the start of recruitment in January 2020, there were no losses and exclusions after randomization, and the analysis was made by the original assigned groups. Baseline demographic data showed a mean age of 63 years (range: 30‐76) and a gender ratio of 55% females and 45% males. iFDPs were evenly allocated in the maxilla and mandible, and most implants had a regular neck configuration (n = 35 RN, n = 5 WN). Overall, 85% of the reconstructions represented free‐end situations and 15% were in edentulous spaces with 2 adjacent teeth. All iFDPs were successfully produced, regardless of the workflow applied. For Test‐1, IOS had to be repeated for 1 study patient due to a corrupted STL file.

### iFDPs – Patient satisfaction / dentist evaluation (primary outcome)

In the double‐blind evaluation of the 3‐unit monolithic ZrO_2_ iFDPs, both the patients and the dentist assigned the highest VAS scores for overall satisfaction for Test‐1, followed by Control, and Test‐2. In general, the mean VAS scores of the dentists were lower than those given by the patients. The highest agreement between patients and dentists was observed for the 3Shape proprietary workflow (Test‐1) with a difference of 21% lower VAS scores given by the dentist compared to patients, followed by Control with a percentage value of 31%, and the second proprietary digital workflow of Dental Wings (Test‐2) with 32%.

**FIGURE 3:**
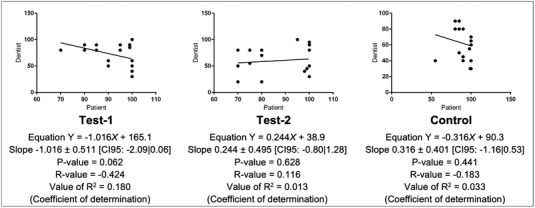
Linear regression analyses for mean VAS scores related to the monolithic ZrO_2_ iFDPs from Test‐1, Test‐2, and Control for overall satisfaction of the patients (x‐axis) and the dentist (y‐axis).

ANOVA analysis for overall patient satisfaction (subjective perception) between Test‐1, Test‐2, and Control showed no significant difference with an f‐ratio value of 0.13 and a corresponding *p*‐value of 0.876. VAS scores representing overall dentist satisfaction (objective assessment) between workflows were also not significantly different, with an f‐ratio value of 1.55 and a p‐value of 0.221. Tables 1 and 2 show the pairwise comparisons of the 3 workflows separately for patient and dentist satisfaction, respectively. Figure 3 displays the regression analyses for overall patient / dentist satisfaction related to the monolithic ZrO_2_ iFDPs from Test‐1, Test‐2, and Control. The relationship between VAS scores of the patient (x‐axis) and the dentist (y‐axis) is weak for all 3 analyses (Fig 3).

**TABLE 1:** Pairwise comparisons of the 3 workflows for overall patient satisfaction (subjective perception) of the monolithic ZrO_2_ iFDPs based on mean VAS scores

Pairwise comparisons Overall Patient satisfaction iFDP (Mean VAS scores)	HSD_0.05_ = 8.45 HSD_0.01_ = 10.66	Q_0.05_ = 3.40 Q_0.01_ = 4.29
Test‐1 vs. Test‐2 T‐1 = 91.40 (SD ± 9.10) T‐2 = 89.60 (SD ± 12.6)	1.80	Q = 0.72 (*p* = 0.866)
Test‐1 vs. Control T‐1 = 91.40 (SD ±9.10) C = 90.35 (SD ±11.34)	1.05	Q = 0.42 (*p* = 0.952)
Test‐2 vs. Control T‐2 = 89.60 (SD ± 12.60) C = 90.35 (SD ± 11.34)	0.75	Q = 0.30 (*p* = 0.975)

HSD = Tukey's honestly significant difference, iFDPs = implant fixed dental prostheses, SD = standard deviation, VAS = visual analogue scale, ZrO_2_ = zirconium dioxide.

**TABLE 2:** Pairwise comparisons of the 3 workflows for overall dentist evaluation (objective assessment) of the monolithic ZrO_2_ iFDPs based on mean VAS scores

Pairwise comparisons Overall Dentist evaluation iFDP (Mean VAS scores)	*HSD_0.05_ = 17.40 HSD_0.01_ = 21.94	Q_0.05_ = 3.40 Q_0.01_ = 4.29
Test‐1 vs. Test‐2 T‐1 = 72.25 (SD ± 21.79) T‐2 = 60.75 (SD ± 26.62)	11.50	Q = 2.25 (*p* = 0.258)
Test‐1 vs. Control T‐1 = 72.25 (SD ± 21.79) C = 61.75 (SD ± 19.62)	10.50	Q = 2.05 (*p* = 0.322)
Test‐2 vs. Control T‐2 = 60.75 (SD ± 26.62) C = 61.75 (SD ± 19.62)	1.00	Q = 0.30 (*p* = 0.990)

HSD = Tukey's honestly significant difference, iFDPs = implant fixed dental prostheses, SD = standard deviation, VAS = visual analogue scale, ZrO_2_ = zirconium dioxide.

### Impressions—Patient perception (secondary outcome)

The 3 sets of 6 questions per patient were separately evaluated for the 3 workflows and are summarized in Table 3. ANOVA analyses revealed significant differences for patients’ perceptions related to the overall level of convenience, bad oral taste, and nausea sensation in favor of the digital techniques, independent of the applied IOS workflow (Test‐1 or Test‐2), over the conventional approach (Control). No statistical differences were evident between both digital impression techniques, whereas Test‐1 had the highest scores in terms of performance rated by the patients in all 6 categories (Table 3).

**TABLE 3:** Questions on patient's perception comparing digital and conventional impression procedures for Test‐1, Test‐2, and Control with means including SD and range of scores [ANOVA]

6 Questions	Test‐1 Digital Impression 3Shape “TRIOS 3”	Test‐2 Digital Impression Dental Wings “Virtuo Vivo”	Control Conventional Impression Polyether “3M Espe Impregum”
What is your opinion on the treatment time for the impression procedure? VAS: unsatisfactory 0–100 excellent	Mean 85.25 (SD ± 13.03) [Range 50–100]	Mean 69.90 (SD ± 21.87) [Range 30–100]	Mean 61.75 (SD ± 29.08) [Range 0–100]
*ANOVA: The f‐ratio value is 5.72. The p‐value is 0.005. The result is significant at p<0.05*. *Pairwise comparisons: Test‐1 vs. Test‐2 (p = 0.084); Test‐1 vs. Control (p = 0.004); Test‐2 vs. Control (p = 0.485)*.			
How convenient was the impression procedure for you? VAS: unsatisfactory 0–100 excellent	Mean 85.90 (SD ± 11.98) [Range 70–100]	Mean 75.50 (SD ± 18.70) [Range 30–100]	Mean 54.00 (SD ± 24.42) [Range 10–90]
*ANOVA: The f‐ratio value is 14.57. The p‐value is < 0.001. The result is significant at p<0.05*. *Pairwise comparisons: Test‐1 vs. Test‐2 (p = 0.204); Test‐1 vs. Control (p<0.001); Test‐2 vs. Control (p = 0.002)*.			
How high was your anxiety level before the impression procedure? VAS: low 0–100 high	Mean 8.25 (SD ± 19.28) [Range 0–70]	Mean 12.25 (SD ± 27.17) [Range 0–100]	Mean 18.00 (SD ± 30.54) [Range 0–100]
*ANOVA: The f‐ratio value is 0.71. The p‐value is 0.499. The result is not significant at p<0.05*. *Pairwise comparisons: Test‐1 vs. Test‐2 (p = 0.879); Test‐1 vs. Control (p = 0.469); Test‐2 vs. Control (p = 0.766)*.			
Was there a bad oral taste present and/or after the impression procedure? VAS: no 0–100 strong sensation	Mean 4.25 (SD ± 10.92) [Range 0–40]	Mean 8.50 (SD ± 16.31) [Range 0–60]	Mean 26.50 (SD ± 23.68) [Range 0–70]
*ANOVA: The f‐ratio value is 8.85. The p‐value is < 0.001. The result is significant at p<0.05*. *Pairwise comparisons: Test‐1 vs. Test‐2 (p = 0.731); Test‐1 vs. Control (p<0.001); Test‐2 vs. Control (p = 0.006)*.			
Did you experience a nausea sensation during impression procedure? VAS: no 0–100 strong sensation	Mean 1.00 (SD ± 4.47) [Range 0–20]	Mean 1.00 (SD ± 3.08) [Range 0–10]	Mean 25.00 (SD ± 31.37) [Range 0–80]
*ANOVA: The f‐ratio value is 11.36. The p‐value is < 0.001. The result is significant at p<0.05*. *Pairwise comparisons: Test‐1 vs. Test‐2 (p = 0.000); Test‐1 vs. Control (p<0.001); Test‐2 vs. Control (p<0.001)*.			
Did you experience pain during impression procedure? VAS: no 0–100 severe pain	Mean 1.00 (SD ± 3.08) [Range 0–10]	Mean 8.50 (SD ± 16.63) [Range 0–60]	Mean 4.85 (SD ± 11.38) [Range 0–40]
*ANOVA: The f‐ratio value is 2.03. The p‐value is 0.141. The result is not significant at p<0.05*. *Pairwise comparisons: Test‐1 vs. Test‐2 (p = 0.118); Test‐1 vs. Control (p = 0.558); Test‐2 vs. Control (p = 0.592)*.			

VAS = visual analogue scale, SD = standard deviation.

## DISCUSSION

The aim of this double‐blind RCT with cross‐over design was to analyze the subjective and objective evaluation of monolithic ZrO_2_ iFDPs in the posterior region. The results showed that the patients were much easier to satisfy than the dentist, regardless of the workflow used. No significant differences were found in patient and dentist evaluations when comparing the 2 proprietary digital workflows (Test‐1, Test‐2) and the conventional approach (Control), respectively. However, the 3Shape workflow consisting of IOS with TRIOS and corresponding lab‐software (Test‐1), consistently received the highest mean scores in all categories from both patients and the dentist. Secondary, all patients favored the digital impression systems (IOS) over the conventional approach, with Test‐1 again achieving the best mean VAS scores. Therefore, the null hypotheses could be confirmed that (1) subjective patient satisfaction was similar when comparing iFDPs in these 3 workflows; (2) patient satisfaction did not correlate to objective dental evaluation; while the hypothesis that (3) patients had no preference upon the impression technique was rejected with IOS being favored.

From the patients' perspective, the applied workflows had demonstrated minor influence on the evaluation of the final implant‐prosthetic reconstructions in the nonesthetic area. This is consistent with results from other RCTs focusing on single‐unit implant crowns, in which patients were similarly satisfied with restorations fabricated completely digitally or in a mixed analog‐digital workflow.^7^, ^27^ A 3‐unit iFDP in the posterior region acts as the occlusal center in the quadrant, it is responsible for the function of 1 chewing side, and influences the appearance by creating the buccal corridor. The impact of a single crown in the posterior region is much lower. Compared to subjective patients’ assessment, the dental operator assessed the restorations according to strict standardized clinical criteria and had a more critical expert view. These differences reflect the need for critical expert assessment, while patient's expectations might be already addressed when the existing tooth space is rehabilitated. The main difference between the two digital workflows, Test‐1 and Test‐2, was the automatically generated design of the lab software. In the present study, Test‐1 iFDPs convinced the clinician with a more streamlined design compared to the Test‐2 workflow; however, the patients judged this difference to be rather negligible. In this context, patients’ judgement is often limited when it comes to the assessment of the quality of the dental therapy, whereas the experts must have high‐quality standards to ensure long‐term stability of restorations.^3^ Here, the question remains: when is the right time to collect patient‐reported data? In the present trial, patients evaluated the monolithic ZrO_2_ iFDPs at the time of prosthesis delivery. Ideally, patients would be interviewed after a certain period of acclimation under everyday conditions in their usual environment. However, in the context of a double‐blind, triple‐armed RCT, this kind of long‐term follow‐up can only be realized at a disproportionately high effort for patients and practitioners. Honest and transparent patient communication from the outset with the alignment of expectations in the context of realistic outcomes is essential for modern patient‐centered dentistry.^23^

The study design with double‐blinding of dentist and patient allows the best possible comparison of human perception of iFDPs. The randomized cross‐over protocol increased the number of iFDPs studied by a factor of 3 to a remarkable total of 60, though only 20 patients were enrolled in this prospective clinical trial. Furthermore, it should be noted that although the 3 workflows were different in terms of the manufacturing process, the final outcome for each group was always a monolithic ZrO_2_ iFDP. Here, the specific trial design allows for direct comparison not influenced by different materials or framework designs. For single‐unit implant crowns, no significant difference in patient satisfaction was found between monolithic and veneered restorations.^17^, ^27^

The clinical transfer of the individual patient situation to the dental technician for the fabrication of a multiunit iFDP can be realized with IOS or polyether impressions.^28^ With the focus on PROMs, the present results have shown that the perception and preference of patients are in favor of digital impression systems over those with plastic materials in a conventional approach. Again, these findings reflect those from the literature in connection with the therapy of implant‐anchored single crowns.^26^, ^29^, ^30^

Clinical trials that focus on patient perceptions are inherently highly subjective. In this context, it should be emphasized that the sample size could be a confounding factor for the overall results in this double‐blind RCT. The number of patients included is relatively small, although the total number of reconstructions could be increased by a three‐arm study design. In addition, digital workflows are subject to extremely rapid changes in their development. Therefore, the results can only reflect the situation at a particular timepoint. Recent developments could change these results. Today, digital treatment and manufacturing processes benefit from open workflows. In this clinical trial, the two digital workflows were company‐specific proprietary workflows, i.e., IOS and laboratory software from the same company. Other combinations could have led to completely different results. Future research needs to focus on PROMs and cost‐effectiveness as routinely implemented outcomes in addition to clinical parameters in order to gain more insights.

Overall, the findings of this RCT underline the importance of understanding patients' expectations in advance of any invasive steps. The dentist's responsibility is rather acting as a medical advisor and moderator based on sound clinical evidence for a personalized solution: objectively, transparently, and in line with the individual patient's need.^1^ In reconstructive dentistry, several therapy options are often conceivable and the need for clarification is particularly important. This is even more pronounced the more patients themselves are involved in financing the dental treatment. Here, a potential bias exists in nationally specific insurance systems, which promote certain forms of therapy—not always congruent with medical appropriateness. Therefore, clinical trials focusing on PROMs help define the patient‐specific minimal standard of care.

## CONCLUSION

In conclusion, the different digital workflows demonstrated minor influence on the subjective and objective evaluation of the monolithic ZrO_2_ iFDPs in the nonesthetic region; however, patients generally favored IOS over conventional impressions.

## CONFLICT OF INTEREST

Tim Joda, Nicola U. Zitzmann and Urs Brägger received lecture fees and research support from the ITI in the past. Aiste Gintaute and Karin Weber do not have any conflicts of interest in regards to the current study.
